# Eribulin activity in soft tissue sarcoma monolayer and three-dimensional cell line models: could the combination with other drugs improve its antitumoral effect?

**DOI:** 10.1186/s12935-021-02337-5

**Published:** 2021-12-04

**Authors:** Javier Escudero, Victoria Heredia-Soto, Yinyin Wang, Patricia Ruiz, Yingying Hu, Alejandro Gallego, Jose Juan Pozo-Kreilinger, Virginia Martinez-Marin, Alberto Berjon, Eduardo Ortiz-Cruz, Daniel Bernabeu, Jaime Feliu, Jing Tang, Andres Redondo, Marta Mendiola

**Affiliations:** 1 Translational Oncology Research Laboratory, Instituto de Investigación del Hospital Universitario La Paz (IdiPAZ), 28046 Madrid, Spain; 2 Center for Biomedical Research in the Cancer Network (Centro de Investigación Biomédica en Red de Cáncer, CIBERONC), Instituto de Salud Carlos III, 28046 Madrid, Spain; 3 Research Program in Systems Oncology, Faculty of Medicine, University of Helsinki, Haartmaninkatu 8, 00290 Helsinki, Finland; 4 Molecular Pathology and Therapeutic Targets Group, Instituto de Investigación del Hospital Universitario La Paz (IdiPAZ), Paseo de la Castellana, 261, 28046 Madrid, Spain; 5 Department of Medical Oncology, Hospital Universitario La Paz, IdiPAZ, Paseo de la Castellana, 261, 28046 Madrid, Spain; 6 Department of Pathology, Hospital Universitario La Paz, IdiPAZ, 28046 Madrid, Spain; 7 Department of Orthopaedic Surgery, Hospital Universitario La Paz, IdiPAZ, 28046 Madrid, Spain; 8 Department of Radiology, Hospital Universitario La Paz, IdiPAZ, 28046 Madrid, Spain; 9 Cátedra UAM-ANGEM, Faculty of Medicine, Universidad Autónoma de Madrid, Paseo de La Castellana, 261, 28046 Madrid, Spain

**Keywords:** Eribulin, Sarcoma, Proliferation, Migration, Invasion, 2D and 3D models

## Abstract

**Background:**

Eribulin has shown antitumour activity in some soft tissue sarcomas (STSs), but it has only been approved for advanced liposarcoma (LPS).

**Methods:**

In this study, we evaluated the effect of eribulin on proliferation, migration and invasion capabilities in LPS, leiomyosarcoma (LMS) and fibrosarcoma (FS) models, using both monolayer (2D) and three-dimensional (3D) spheroid cell cultures. Additionally, we explored combinations of eribulin with other drugs commonly used in the treatment of STS with the aim of increasing its antitumour activity.

**Results:**

Eribulin showed activity inhibiting proliferation, 2D and 3D migration and invasion in most of the cell line models. Furthermore, we provide data that suggest, for the first time, a synergistic effect with ifosfamide in all models, and with pazopanib in LMS as well as in myxoid and pleomorphic LPS.

**Conclusions:**

Our results support the effect of eribulin on LPS, LMS and FS cell line models. The combination of eribulin with ifosfamide or pazopanib has shown in vitro synergy, which warrants further clinical research.

**Supplementary Information:**

The online version contains supplementary material available at 10.1186/s12935-021-02337-5.

## Background

Soft tissue sarcomas (STSs) represent approximately 1% of all adult cancers. This heterogeneous group of tumours is currently subclassified by the World Health Organization (WHO) into approximately 80 histological subtypes [1]. From a genetic point of view, STSs are classified into 2 categories: those considered to harbour simple genetic alterations and those with complex karyotypes. Liposarcoma (LPS), one of the most common STSs, has been classically subclassified into 4 subtypes: well-differentiated (WDLPS), dedifferentiated (DDLPS), myxoid (MLPS) and pleomorphic (PLPS), although the most recent WHO classification has included a fifth subtype: myxoid pleomorphic LPS [2]. WDLPS and DDLPS together comprise the largest subgroup of liposarcomas, constituting a histologic and behavioural spectrum of one disease, and sharing a genetic alteration: the amplification of the chromosomal region 12q14-15, which involves the *MDM2* and *CDK4* genes [3]. MLPS is characterised by the presence of a recurrent translocation, t(12;16)(q13;p11), with the implication of *FUS* and *DDIT3* genes, and PLPS has no characteristic molecular alteration [4]. Leiomyosarcoma (LMS) is characterised by high chromosomal instability [5–7]. Finally, adult fibrosarcoma (FS) shows multiple nonspecific chromosomal abnormalities [8].

Treatment of advanced STS is mainly based on chemotherapy and other systemic treatments, although surgery and radiotherapy can also be used with palliative intention in some patients. Doxorubicin is usually administered as first-line treatment. Other drugs, such as ifosfamide, trabectedin, pazopanib, eribulin, gemcitabine (in combination) and dacarbazine are options for subsequent treatment lines [9]. Additionally, in WDLPS and DDLPS, palbociclib has shown promising clinical activity [10].

Eribulin is an antitumour agent acting mainly as a microtubule dynamics inhibitor. Other mechanisms of action have also been proposed, such as vascular remodelling, reversion of the epithelial to mesenchymal transition (EMT) and suppression of migration and invasion [11–14]. Eribulin clinical activity was initially assessed in a phase II trial of patients with STS with several histologies, including LPS, LMS, synovial sarcoma and a mixed group of less common STSs [15]. Based on the higher activity level in LPS and LMS subtypes, a phase III trial was developed to compare eribulin with dacarbazine in pre-treated patients with these two histologies. This study achieved its primary endpoint, showing a benefit in overall survival (OS) for eribulin. However, regulatory agencies only approved eribulin for advanced LPS based on the results of the subgroup analysis, which did not demonstrate an OS benefit for the LMS cohort [16].

The rarity of sarcomas makes it difficult to design clinical trials or make clinical decisions regarding specific subtypes. In this context, cellular models are a valuable approach, particularly for drug testing studies, which can provide useful preclinical data to help with the design of further clinical research. Most current studies are performed on monolayer two-dimensional (2D) cell models, which are still the most commonly used due to their high standardisation and cost effectiveness. However, they have a number of limitations, particularly important for drug studies. The main disadvantage is that cells are cultured on flat dishes, where there is no spatial complexity, and they are all equally exposed to compounds, which is not representative of a real cancer cell environment [17]. This scenario has led to the emergence of three-dimensional (3D) models, including spheroids, which more faithfully represent the tumour’s phenotypic characteristics and mimic its structure [18].

In this study, we explored the activity of eribulin in a STS cell line panel: LPS and LMS, which were the target of the phase III clinical trial, and FS, a rare sarcoma, in which eribulin was previously tested in vitro and in xenografts [19]. We investigated the effect on proliferation, migration and invasion capabilities, in 2D and 3D conditions. Finally, we studied the possible synergistic effect of eribulin with other drugs commonly used in the treatment of STS.

## Methods

### Lines, compounds and cell culture

LIPODL221 (MLPS), LPS224 (DDLPS) and LPS246 (DDLPS) cell lines were obtained from the MD Anderson biobank core facility. SW872 (PLPS), SK-UT-1 (LMS), HT1080 (FS) and 93T449 (WDLPS) were kindly provided by Dr.Carnero from the Biomedicine Institute (Seville, Spain). The cell lines were tested routinely for mycoplasma, and authenticated by genetic profiling using polymorphic short tandem repeat loci from the Geneprint 10 kit (Promega, USA).

All culture media were supplemented with a concentration of 10% foetal bovine serum (v/v), 1% L-glutamine (v/v) and antibiotics (100 units/ml of penicillin and 100 µg/ml of streptomycin), all from Merck (Germany). SK-UT-1 (LMS), SW872 (PLPS), LIPODL221 (MLPS), LPS224 (DDLPS) and LPS246 (DDLPS) were cultured in DMEM, SW872 (PLPS) supplemented with 0.01% sodium pyruvate (PyrNa); and SK-UT-1 (LMS) supplemented with 0.01% non-essential amino acids (NEAA) + 0.001% 4-(2-hydroxyethyl)-1-piperazineethanesulfonic acid (HEPES) + 0.001% pyruvate. HT1080 (FS) and 93T449 (WDLPS) were grown in F-10 medium. Cultures were maintained at 37 ºC in a humid atmosphere and 5% CO_2_. Cells were regularly tested for mycoplasma infection. All media and supplements were purchased from Sigma-Aldrich (USA).

Eribulin was kindly provided by Eisai Inc. (USA). Doxorubicin, ifosfamide, gemcitabine, trabectedin, pazopanib and palbociclib were obtained from Selleckchem (UK).

### Proliferation and cell cycle assyas

The monolayer experiment cells were seeded in 96-well plates (MW96) (Corning, USA) at a variable density, previously established for each cell line (detailed information included in Additional file 1: Table S1). Cellular confluence was measured at various time points by sulphorhodamine B (SRB) staining, as previously described [20]. Absorbance was measured at 564 nm on a Synergy 4 spectrophotometer using the Gen5 program (BioTek, USA).

For 3D experiments, cells were seeded in ultra-low attachment (ULA) round bottom plates The optimal spheroid size relies on the specific assay requirements, but is usually within a 200–500 µm diameter range We established a pre-set density for each cell line, to obtain a spheroid diameter around 300 µm at day 4, as previously described in ULA plates [21, 22]. Growth was tracked measuring spheroid diameter at days 4, 7, 10 and 14. Image acquisition and analyses were performed with a Celigo S plate cytometer (Nexcelom, USA).

The drug screening experiments were performed in MW96 plates. The design of the plate was as follows: all border wells contained phosphate buffered saline (PBS), to avoid evaporation issues. The rest of the plate contained the pre-set number of cells for each cell line and condition. With this design, 9 columns with 6 replicates each will be exposed to increasing concentrations of the explored drug, and one column will be used as control. For monolayer cultures, cells were exposed 24 h after seeding to 1:2 dilutions of drug concentrations (detailed in Additional file 1: Table S1) of eribulin and the other tested drugs (doxorubicin, ifosfamide, gemcitabine, trabectedin, pazopanib and palbociclib) for 72 h. Inmediately after, cell viability was measured by SRB staining as was performed for the proliferation assays. For the 3D experiments, after 4 days of culture, the spheroids were exposed to 1:2 dilutions of drug concentrations, for 72 h. Cell viability was measured after this period using a CellTiter-Glo Luminescent assay (Promega) according to the manufacturer’s protocol and bright field images were taken. At least two experiments were performed for each cell line.

We calculated 2D and 3D growth inhibitory concentration by 50% (GI_50_) using non-linear regression with GraphPad Prism 7 software (GraphPad Software, USA).

For cell cycle and apoptosis assays, cells were seeded as described previously for proliferation assays (Additional file 1: Table S1), and exposed, 24 h after seeding, to their corresponding eribulin GI_50_ concentrations (Table 1) for 72 h. For analysis, cells are previously fixed with 70% ethanol and nuclei were stained with propidium iodide (Merck). The distribution of integrated intensity was visualized and quantified by the Celigo S plate cytometer (Nexcelom) gating tool, as counts and percentage over the total cell count. Experiments were performed twice and including at least two replicates.

**Table 1:** Eribulin 2D and 3D GI_50_ values (nM). Concentration is displayed as ± standard deviation. 3D/2D ratio is also shown

Cell line	Sarcoma subtype	Eribulin GI_50_ 2D	Eribulin GI_50_ 3D	GI_50_ 3D/ GI_50_ 2D
HT1080	FS	5.07 ± 2.37	4.67 ± 1.75	0.92
SK-UT-1	LMS	0.17 ± 0.04	0.39 ± 0.13	2.29
SW872	PLPS	2.18 ± 0.18	3.54 ± 2.49	1.62
LIPODL221	MLPS	1.03 ± 0.22	> 100	NE
93T449	WDLPS	1.24 ± 0.17	47.93 ± 16.92	38.65
LPS224	DDLPS	11.75 ± 0.86	> 100	NE
LPS246	DDLPS	1.25 ± 0.12	> 100	NE

*FS* Fibrosarcoma, *LMS* Leiomyosarcoma, *PLPS* Pleomorphic liposarcoma, *MLPS* Mixoid liposarcoma, *WDLPS* Well-differentiated liposarcoma, *DDLPS* Dedifferentiated liposarcoma, *NE* Not evaluable

### 2D and 3D migration and invasion assays

We employed 8-μm pore transwell inserts for migration (Ref.354578, Corning), and Matrigel coated inserts for invasion experiments (Ref. 354480, Corning). The insert was fixed and stained 24 h after seeding using the Diff-Quick method (QCA, Spain). Two different fields were photographed at 20 × magnification for the subsequent cell count using the ImageJ program (National Institutes of Health, USA).

For the study of migration and invasion capacity in 3D, 4 day spheroids were placed on a previously solidified Matrigel layer for migration, or embedded in Matrigel for invasion assays.. The images were taken and quantified after 3 to 7 days using ImageJ (NIH).Two experiments were performed and the measures obtained in two fields are represented as a ratio with the non-treated control cells.

### Establishment of eribulin-resistant cell lines and exploratory antibody array analysis

Eribulin-resistant cell line was established from 93T449 LPS cells by continuous exposure to eribulin (GI_50_ value, 1.24 nM) over a period of three months.

Semi-quantitative detection of 1000 human proteins was performed using the RayBio C-series Human Cytokine Antibody Array C1000 (RayBiotech, USA) following manufacturer's instructions. Dots detection was performed with the GenePix 4100A Microarray scanner and quantified by GenePix Pro 7 Software System (Molecular Devices, USA). The raw numerical intensity data were extracted and subjected to background subtraction before normalising the signal for each cytokine against the positive control signals in each array. Duplicate discordant results were eliminated for the analysis. A fold change over 2 times and pvalues < 0.05 were used as cutoff.

### Drug combinations

Drug combination studies were performed in pairs, combining eribulin with the other drugs. Treatment was established for 72 h, with a maximum concentration up to 10 mM (specific concentrations for each cell line are based on single drug experiments and summarized in Additional file 1: Table S1), and 1:3 dilutions. According to a 6 × 6 dose–response matrix design, both, the effect of the single drugs, as well as the combination, were tested. Experiments were first analysed using Synergy-Finder R package (http://bioconductor.org/packages/release/bioc/html/synergyfinder.html) [23, 24].

We used 4 available algorithms to test the synergy between drugs: the Bliss model (BLISS), Highest Single Agent (HSA), Loewe additivity model (LOEWE) and Zero Interaction Potency (ZIP) [25]. The effect was considered synergistic only when the 4 algorithms produced a positive scores [26]. Additionally, to evaluate the overall efficacy of a drug combination, drug combination sensitivity scores were determined by using a Combination Sensitivity Score (CSS) approach, which is based on the area under their dose–response curves at relative GI_50_ values of compounds [27]. A top drug combination was assumed only if all the synergy scores had a positive value and CSS was > 60. Experiments for each combination were performed at least two times.

### Statistical analysis

The averages and standard deviations of the data, as well as the Student T tests, were performed using Microsoft Office Excel 2007 (Microsoft, USA). As previously indicated, GI_50_ calculation was performed by non-linear interpolation using GraphPad Prism 7 software (GraphPad Software).

## Results

### Cell characterisation

**Fig. 1:**
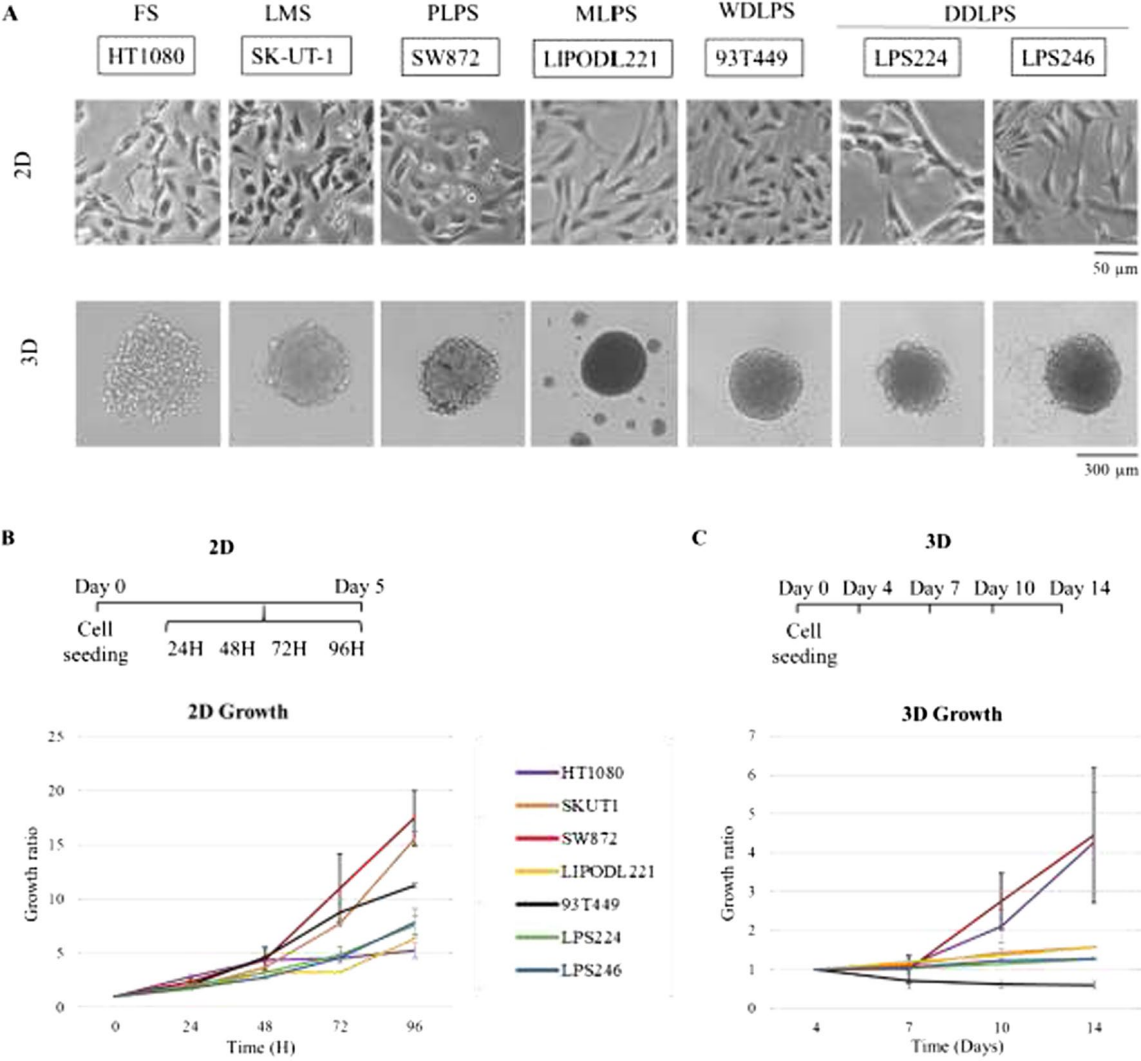
Basal morphology and growth of cell lines tested. **A** Bright field micrographs for 2- and 3D culture. **B** 2D growth and C. 3D growth, including experiment schedules and growth curves. Each cell line growth is normalised to seeded cells at time 0 for 2D culture, and to day 4 spheroid diameter for 3D

The morphology of the cells is shown in Fig. 1A. All cell lines grew with a mesenchymal morphology under 2D culture. Regarding 3D, SK-UT-1 (LMS) and all the LPS cell lines grew as compact spheroids. LIPODL221 (MLPS) had the peculiarity of growing as multispheres. HT1080 (FS) formed less compact spheroids. The experimental schedule and growth rates are illustrated in Fig. 1B, C, showing that SW872 appears to growth faster compared to the other cell lines, under 2D and 3D conditions, although it does not reach statistical significance.

### Effect of eribulin and other drugs on proliferation

The most sensitive cell line to eribulin in 2D and 3D conditions was SK-UT-1 (LMS). HT1080 (FS) and SW872 (PLPS) keep similar values of sensitivity in both conditions, while the rest of the cells showed increases in their GI_50_ when grown in 3D conditions. Values in 2D were in the nanomolar range for all cell lines, and also for 4 of them in 3D conditions. The other 3 lines did not reach their GI_50_ values with a 100 nM exposure (Table 1). Examples of growth curves under eribulin exposure of each line, as well as a representation of GI_50_ values can be seen on Additional file 2: Fig. S1.

Additionally, eribulin promoted morphological changes in all lines except LPS246 (DDLPS), changing their basal mesenchymal morphology to a more rounded one (Additional file 3: Fig. S2).

The effect of other drugs was also tested on the cell lines. The cells were exposed to various drug concentrations (up to 10 mM). Pazopanib and palbociclib presented a GI_50_ at a micromolar range. Doxorubicin, gemcitabine and trabectedin presented GI_50_ at a nanomolar range, and ifosfamide presented GI_50_ at a millimolar range (Additional file 4: Table S2).

### Effect of eribulin on cell cycle

Cell cycle and apoptosis were also explored by DNA content quantification of cells. A general increase in the percentage of cells in sub G0 and S-phase was observed in all cell lines, indicating that eribulin treatment led to apoptosis and S-phase arrest. Regarding checkpoints, a decreased G0/G1-phase was observed, and G2/M-phase variations seemed to be dependent on the cell line (Additional file 5: Fig. S3).

### Effects of eribulin on migration and invasion in both 2D and 3D cultures

**Fig. 2:**
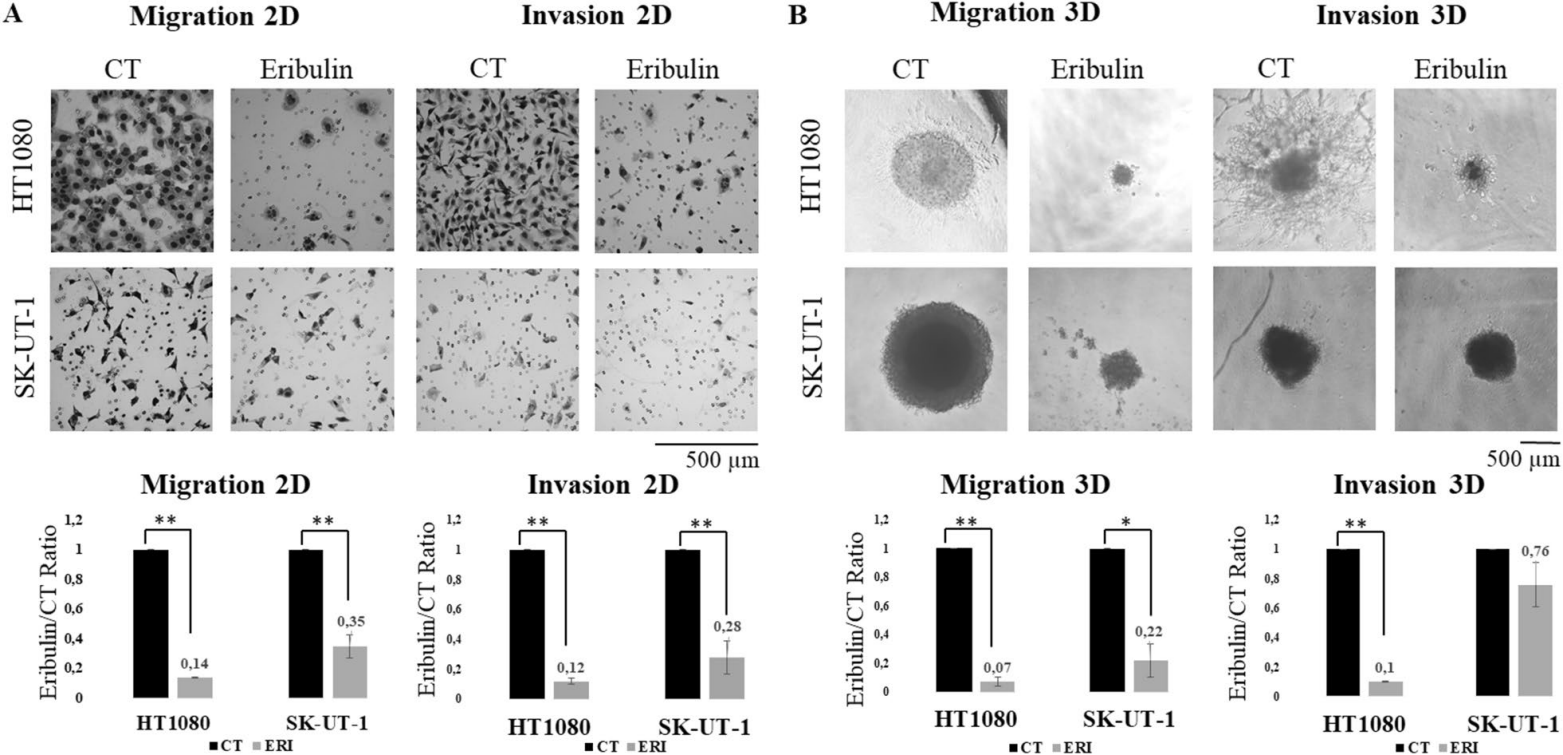
Eribulin effect on migration and invasion in FS and LMS cell lines in both 2D and 3D cultures. **A** Micrographs for 2D of a representative experiment of HT1080 and SK-UT-1 (upper panel) and bar plot for quantification of eribulin-treated cells vs control (lower panel). **B** Micrographs for 3D of a representative experiment of HT1080 and SK-UT-1 (upper panel) and bar plot for quantification of eribulin-treated cells vs control (CT) nontreated cells (lower panel). *p < 0.05, **p < 0.005

In the LMS and FS cell lines, an overall decrease in migration and invasion was observed. Migration decreased between 40 and 90%, and invasion was abolished in 20% to 90%. The effects were greater in 3D than in monolayer cultures. A representative experiment is shown in Fig. 2.

However, decreased migration and invasion was less visible in LPS cell lines, and not statistically significant. Some cells did not migrate or invade in basal conditions (LIPODL221, MLPS), whereas others (LPS224 and LPS246, both DDLPS), showed a high increase in their GI_50_ values in 3D, so they were as well excluded from this part. In the 2 remaining lines, 93T449 and SW872, only a modest decrease in both properties was observed (data not shown).

### Exploratory identification of pathways implicated in eribulin action

We next sought to determine potential action modes for eribulin. To do so, we used 93T449 LMS cell line to generate an eribulin-resistant line through continuous drug exposure. An exploratory analysis of deregulated proteins included and enriched pathways, pointed candidate pathways to explore, as is protein metabolism (Additional file 6: Fig. S4).

### Effect of eribulin combinations in 2D cultures

Combinations of eribulin and a second drug were studied to establish potential synergies. We selected drugs commonly used in STS (doxorubicin, ifosfamide, trabectedin, pazopanib and gemcitabine) plus palbociclib, as possible agents for LPS.

**Fig. 3:**
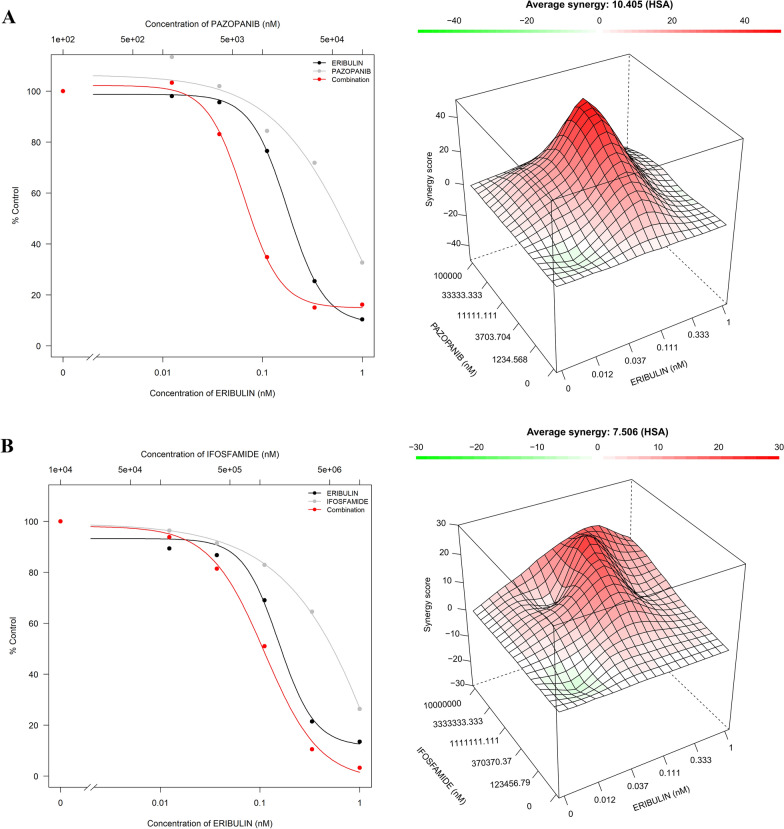
Effect of synergistic combinations in cell growth. SK-UT-1 cell line plots are presented as example. **A** Eribulin plus pazopanib. **B** Eribulin plus ifosfamide. Representative experiments are shown. First plot represents the drug effect on cell proliferation, as follows: grey line for ifosfamide or pazopanib alone, black line for eribulin, and red line for combinations. Second plot corresponds to the 3D synergy map obtained by HSA algorithm with SynergyFinder 2.0 software. Red colours represents synergy areas and green colours represent non-synergistic areas

Of all the tested combinations, 2 showed synergy using our restrictive criteria (a positive score in all 4 algorithms) with SynergyFinder (http://synergyfinder.org). First, the combination of eribulin and pazopanib was synergistic for SK-UT-1 (LMS), SW872 (PLPS) and LIPODL221 (MLPS) cell lines (Fig. 3A and Table 2). Second, and more impressively, the combination of eribulin and ifosfamide showed consistent synergistic action in all the cell lines tested (Fig. 3B and Table 2). These results were confirmed with Tidycomb, with the exception of the LIPODL221 (MLPS) cell line (Additional file 7: Fig. S5).

**Table 2:** Combination scores for eribulin-ifosfamide and eribulin-pazopanib calculated by Synergy finder. Mean of two experiments and SD are included for each algorithm

Cell line	Combination	ZIP	HSA	BLISS	LOEWE
HT1080	Eribulin + Ifosfamide	7.27 ± 2.41	10.08 ± 1.94	8.22 ± 2.37	4.39 ± 1.29
SK-UT-1		3.74 ± 0.76	7.07 ± 1.26	4.18 ± 0.84	0.7 ± 2.63
SW872		7.49 ± 4.24	8.75 ± 5.07	7.8 ± 4.32	4.48 ± 4.31
LIPODL221		1.31 ± 1.55	6.07 ± 1.33	2,90 ± 0.34	1.99 ± 0.50
93T449		5,19 ± 0.28	7.74 ± 2.48	6.48 ± 2.38	2.55 ± 1.61
LPS224		6,98 ± 0.04	7.77 ± 0.99	7.81 ± 0.44	4.26 ± 0.34
LPS246		2.27 ± 1.15	5.28 ± 0.17	2.68 ± 1.25	1.69 ± 0.32
SK-UT-1	Eribulin + Pazopanib	4.08 ± 4.34	10.25 ± 0.50	5.17 ± 2.49	5.53 ± 1.31
SW872		8.81 ± 1.92	13.97 ± 0.03	9.19 ± 1.41	8.00 ± 0.03
LIPODL221		3.38 ± 2.29	11.07 ± 1.40	3.37 ± 2.24	7.67 ± 1.98

*ZIP* Zero Interaction Potency, *HSA* Highest Single Agent, *LOEWE* Loewe additivity model, *BLISS* Bliss model

## Discussion

Fewer new therapies or drug combinations with clinically relevant efficacy have been identified for STS than for other tumours, in part because these tumours occur less frequently and are highly heterogeneous. Consequently, most clinical trials have included all (or many) STS subtypes together. These entities are different from a histological and molecular point of view, and they do not necessarily share the same drug sensitivity [28].

Cellular models are a useful tool with which to perform drug testing, especially in rare entities such as STS, given that they can serve as the basis for new clinical trial designs. Traditionally, the most commonly performed experiments have been based on cell proliferation or apoptosis in 2D conditions. Currently, we can analyse the effect of drug exposure on other aspects that are important for cancer progression, such as migration and invasion. Moreover, we already know that the classical monolayer culture, although it has a low cost and is easy to handle, does not reflect the architectural situation in the tumour. Spheroids are tridimensional structures that mimic the spatial architecture of tumour cells, and they more accurately represent the pattern of drug diffusion as it actually occurs in the tumour from the surrounding vasculature [18]. Although various techniques have been proposed for 3D culture experiments, the use of non-adherent plates is particularly interesting for drug testing. These plates work in high throughput conditions, with an elevated number of replicates, all with the same size, and allow the study not only of proliferation, but also migration and invasion [21, 22]. We have employed all of these approaches to better characterise the action of eribulin in these cell lines.

Eribulin has been previously studied in sarcomas in 2D culture models. There are positive data with preclinical models of Ewing's sarcoma, rhabdomyosarcomas and osteosarcoma cell lines [29]. Regarding the subtypes examined in this study, eribulin has been reported to exert an antiproliferative effect and to induce apoptosis in primary and established DDLPS lines grown in monolayer. An effect on cell growth in cell lines corresponding to LMS (SK-L-MS1) and FS (HT1080) has also been observed. Similarly, in our study eribulin also produced an inhibitory effect on cell growth in LPS, LMS and FS cell lines [30–32].

A modification of cell morphology was observed in the majority of cell lines, which is consistent with one of the mechanisms of action of eribulin. This mechanism involves the suppression of microtubule dynamics and EMT, which can prompt changes in cell morphology [33]. These changes have also been observed in other cellular models, and particularly in LPS and LMS cell lines, it has been related to cellular differentiation [34, 35]. Effects on apoptosis and cell cycle have been also reported, and associated to apoptosis and mitotic arrest in osteosarcoma and breast cell lines [36, 37]. In our models, eribulin led to significantly increased apoptosis rate and S-phase arrest, while G2/M arrest seemed to be dependent on each cell line.

There have been few studies of the effect of eribulin on 3D STS cell models. A previous report with a 3D LM8 osteosarcoma model suggested an impact on proliferation [38]. Eribulin’s action has not been explored previously under 3D conditions in LMS, FS or LPS, nor has it been compared with 2D. While the HT-1080 FS cell line maintained similar GI_50_ values as the monolayer culture, the LPS and LMS models showed an increase in their GI_50_ values, primarily to nanomolar values, and even to a micromolar range in some cases. This increase could be due to the spheres’ architecture, given that HT-1080 had a less compact structure. A loose aggregate is more similar to a 2D culture, in which all cells are equally exposed to oxygen, nutrients and drugs. The rest of the cell lines, which had a more compact structure, were hindered by a diffusion gradient through the inner layers. They probably required a subsequent increase in the GI_50_ values, given that not all the cells were equally exposed.

Additionally, in compact spheroids, only the outer ring proliferates, while the rest of the cells are quiescent, and some drugs need proliferation of cells for their activity. These findings could favour an increase in GI_50_ values that would better represent the situation in the tumour [22, 39]. Differences in 2D and 3D sensitivities have been reported previously in sarcomas, which generally grow in a very tight structure in 3D. For example, chondrosarcoma cell line monolayers sensitive to doxorubicin displayed a high level of resistance under 3D conditions [40].

Eribulin has been reported to affect migration and invasion of breast cancer cell lines in 2D conditions [13]. It has also been reported to inhibit migration in scratch assays in primary WDLPS and DDLPS lines. We achieved similar results in these subtypes, but no effect was observed on the PLPS cell line subtype. The most impressive results were observed in the LMS and FS cell lines, with an important decrease in migration and invasion in 2D and 3D conditions. There are no previous reports on this finding [32]. These findings are consistent with the morphological changes observed under eribulin exposure involving a mesenchymal to epithelial switch, which could be related to a decrease in migration and invasion on STS models [35].

LPS is one sarcoma subtype were eribulin has been approved and the understanding of resistance mechanisms is increasingly important. Some mechanisms has been already implicated, as the activation of PI3K [41]. We have generated a resistant cell line from the parental 93T449 by eribulin exposure and we have explored dysregulated molecules under a pathway enrichment approach. Our findings suggest that protein metabolism is an over-represented process in this resistant cell line. Nowadays is considered that cellular metabolic rewiring is essential for tumor development, and that metabolic alterations are related to sensitivity (or resistnace) of chemotherapeutics agents. Our preliminary findings suggest that this process could be implicated in Eribulin response, although further experiments will be required to confirm this data [42].

Once the effects of eribulin were observed, studying combinations of eribulin with other drugs commonly used in STS became an attractive option. Few combinations have been previously explored in STS cell line models [32, 43]. Regarding eribulin combinations, a recent study was published suggesting activity with an AKT inhibitor (MK-2206) in FS and LMS cell lines [31].

Synergy indicates that 2 drugs combined exert a greater effect than that expected from the 2 drugs individually. In vitro studies are the basis for rational drug combination experiments, but they are not easy to evaluate and translate into clinical use [44]. Many methods have been developed to evaluate synergy between drugs in vitro, and using more than 1 scientific evaluation method should provide more consistent results. In our study, we have explored the combination of eribulin with a complete panel of drugs commonly used in STS with restrictive synergy requirements. All of the 4 scores obtained by the Synergy Finder analysis had to show a positive value, whereas other studies usually consider synergy to have occurred when only one of the algorithms is positive [45, 46]. Our approach helped us obtain greater certainty in the results.

We found 2 interesting combinations. First, eribulin and pazopanib appeared to be synergistic for LMS, MLPS and PLPS, but not for other LPSs. Pazopanib is a potent and selective multitargeted receptor tyrosine kinase inhibitor that showed efficacy in a phase III trial in STS, and it is currently approved for the treatment of advanced STS (except LPS) pretreated with anthracyclines [47, 48]. Additionally, the combination of eribulin and ifosfamide was consistently synergistic in all lines and subtypes tested. Ifosfamide is an alkylating agent classically used in neo/adjuvant treatment or for first line treatment of advanced STS in combination with an anthracycline. It is also used at high doses as monotherapy in second-line treatment of STS. Due to the consistency of results among the STS subtypes analysed in our study, this combination needs further exploration. From a clinical point of view, the eribulin-pazopanib combination would need careful investigation due to the toxicity profile of each drug. In addition, ifosfamide is associated with high haematological toxicity, which could be increased with eribulin. Therefore, a hypothetical phase I clinical trial to explore this combination should start with a low dose of ifosfamide.

## Conclusions

We have proved eribulin’s effect on proliferation, migration and invasion in STS cell line models. The preclinical results we have obtained are sufficiently robust to consolidate its antitumour effect in this context. Additionally, the combination of eribulin with pazopanib or ifosfamide has a synergistic effect in STS, which warrants further clinical research.

## Supplementary Information

**Additional file 1:** **Table S1. **Detailed experimental conditions for employed cell lines, covering seeding densities and drug dose ranges for 2- and 3D conditions.

**Additional file 2: Figure S1. **Eribulin dose response curves for cell lines studied. A representative experiment is shown for A) 2D and B) 3D culture conditions. Red line represents the value for GI_50_ values. C) GI_50_ Scatter plots. Cells haves been split in two plots according to their values for a better representation. In 3D experiments, only 4 cell lines are presented since the other 3 are over the tested drug range, and considered as resistant.A representative experiment is shown for A) 2D and B) 3D culture conditions. Red line represents the value for GI_50_ values. C) GI_50_ Scatter plots. Cells have been split in two plots according to their values for a better representation. In 3D experiments, only 4 cell lines are presented since the other 3 are over the tested drug range, and considered as resistant.

**Additional file 3: Figure S2.** Eribulin effect on morphology. Phenotypical changes of cell lines exposed to eribulin for 72h.

**Additional file 4: Table S2. **2D GI_50_ values for studied drugs. The concentration value is displayed as mean ± standard deviation.

**Additional file 5: Figure S3.** Effect of eribulin in Cell cycle. Bar plots show Cell cycle distribution for subG0, G0/G1, S and G2/M phases comparison in untreated and eribulin exposed cells. *p<0.05, **p<0.005.

**Additional file 6: Figure S4.** Pathway enrichment analysis for disregulated processes between parental and resistant 93T449. NES: Normalized enrichment score; pval: p value.

**Additional file 7: Figure S5.** Tidycomb Analysis. Eribulin combination with A. Ifosfamide, B. Pazopanib and C. Doxorubicin, on LMS and LPS cell lines. The area highlighted in red corresponds to the score values considered as indicators of synergy.

## Data Availability

Data are available from the corresponding authors on reasonable request.

## Abbreviations

BLISS: Bliss model algorithm
CSS: Combination Sensitivity Score
DDLPS: Dedifferentiated liposarcoma
DMEM: Dulbecco’s Modified Eagle Medium
EMT: Epithelial to mesenchymal transition
FS: Fibrosarcoma
GI50: Growth Inhibitory Concentration by 50%
HEPES: 4-(2-Hydroxyethyl)-1-piperazineethanesulfonic acid
HSA: Highest Single Agent algorithm
LMS: Leiomyosarcoma
LOEWE: Loewe additivity model algorithm
LPS: Liposarcoma
MLPS: Myxoid liposarcoma
MW96: 96-Well plates
NEAA: Non-essential amino acids
OS: Overall survival
PLPS: Pleomorphic liposarcoma
PyrNa: Sodium pyruvate solution
SRB: Sulphorhodamine B
STS: Soft tissue sarcomas
ULA: Ultra-low attachment
WDLPS: Well-differentiated liposarcoma
ZIP: Zero Interaction Potency algorithm

## References

[1] Kallen ME, Hornick JL (2021). The 2020 WHO classification: what's new in soft tissue tumor pathology?. Am J Surg Pathol.

[2] Fletcher C BJ, Anonescu C, Merens F: WHO classification of tumours: soft tissue and bone tumours, Vol. 3, 5th ed. WHO Classification of Tumours Editorial Board; 2020. ISBN: 978-92-832-4502-5. https://publications.iarc.fr/Book-And-Report-Series/Who-Classification-Of-Tumours/Soft-Tissue-And-Bone-Tumours-2020

[3] Thway K (2019). Well-differentiated liposarcoma and dedifferentiated liposarcoma: an updated review. Semin Diagn Pathol.

[4] Anderson WJ, Jo VY (2019). Pleomorphic liposarcoma: updates and current differential diagnosis. Semin Diagn Pathol.

[5] Serrano C, George S (2013). Leiomyosarcoma. Hematol Oncol Clin North Am.

[6] George S, Serrano C, Hensley ML, Ray-Coquard I (2018). Soft tissue and uterine leiomyosarcoma. J Clin Oncol.

[7] Mangla A, Yadav U. Leiomyosarcoma. In: StatPearls [Internet]. Treasure Island (FL): StatPearls Publishing; 2021. https://www.ncbi.nlm.nih.gov/pubmed/31869131

[8] Toro JR, Travis LB, Wu HJ, Zhu K, Fletcher CD, Devesa SS (2006). Incidence patterns of soft tissue sarcomas, regardless of primary site, in the surveillance, epidemiology and end results program, 1978–2001: an analysis of 26,758 cases. Int J Cancer.

[9] Casali PG, Abecassis N, Aro HT, Bauer S, Biagini R, Bielack S, Bonvalot S, Boukovinas I, Bovee JVMG, Brodowicz T (2018). Soft tissue and visceral sarcomas: ESMO-EURACAN clinical practice guidelines for diagnosis, treatment and follow-up. Ann Oncol.

[10] Dickson MA, Schwartz GK, Keohan ML, D'Angelo SP, Gounder MM, Chi P, Antonescu CR, Landa J, Qin LX, Crago AM (2016). Progression-free survival among patients with well-differentiated or dedifferentiated liposarcoma treated with CDK4 inhibitor palbociclib: a phase 2 clinical trial. JAMA Oncol.

[11] Smith JA, Wilson L, Azarenko O, Zhu X, Lewis BM, Littlefield BA, Jordan MA (2010). Eribulin binds at microtubule ends to a single site on tubulin to suppress dynamic instability. Biochemistry.

[12] Funahashi Y, Okamoto K, Adachi Y, Semba T, Uesugi M, Ozawa Y, Tohyama O, Uehara T, Kimura T, Watanabe H (2014). Eribulin mesylate reduces tumor microenvironment abnormality by vascular remodeling in preclinical human breast cancer models. Cancer Sci.

[13] Yoshida T, Ozawa Y, Kimura T, Sato Y, Kuznetsov G, Xu S, Uesugi M, Agoulnik S, Taylor N, Funahashi Y (2014). Eribulin mesilate suppresses experimental metastasis of breast cancer cells by reversing phenotype from epithelial-mesenchymal transition (EMT) to mesenchymal-epithelial transition (MET) states. Br J Cancer.

[14] Swami U, Shah U, Goel S (2015). Eribulin in cancer treatment. Mar Drugs.

[15] Schöffski P, Ray-Coquard IL, Cioffi A, Bui NB, Bauer S, Hartmann JT, Krarup-Hansen A, Grünwald V, Sciot R, Dumez H (2011). Activity of eribulin mesylate in patients with soft-tissue sarcoma: a phase 2 study in four independent histological subtypes. Lancet Oncol.

[16] Schöffski P, Chawla S, Maki RG, Italiano A, Gelderblom H, Choy E, Grignani G, Camargo V, Bauer S, Rha SY (2016). Eribulin versus dacarbazine in previously treated patients with advanced liposarcoma or leiomyosarcoma: a randomised, open-label, multicentre, phase 3 trial. Lancet.

[17] Lamhamedi-Cherradi SE, Santoro M, Ramammoorthy V, Menegaz BA, Bartholomeusz G, Iles LR, Amin HM, Livingston JA, Mikos AG, Ludwig JA (2014). 3D tissue-engineered model of Ewing's sarcoma. Adv Drug Deliv Rev.

[18] Colella G, Fazioli F, Gallo M, De Chiara A, Apice G, Ruosi C, Cimmino A, de Nigris F (2018). Sarcoma spheroids and organoids-promising tools in the era of personalized medicine. Int J Mol Sci.

[19] Towle MJ, Nomoto K, Asano M, Kishi Y, Yu MJ, Littlefield BA (2012). Broad spectrum preclinical antitumor activity of eribulin (Halaven(R)): optimal effectiveness under intermittent dosing conditions. Anticancer Res.

[20] Orellana EA, Kasinski AL (2016). Sulforhodamine B (SRB) assay in cell culture to investigate cell proliferation. Bio Protoc.

[21] Vinci M, Gowan S, Boxall F, Patterson L, Zimmermann M, Court W, Lomas C, Mendiola M, Hardisson D, Eccles SA (2012). Advances in establishment and analysis of three-dimensional tumor spheroid-based functional assays for target validation and drug evaluation. BMC Biol.

[22] Heredia-Soto V, Redondo A, Kreilinger JJP, Martínez-Marín V, Berjón A, Mendiola M (2020). 3D culture modelling: an emerging approach for translational cancer research in sarcomas. Curr Med Chem.

[23] Ianevski A, He L, Aittokallio T, Tang J (2017). SynergyFinder: a web application for analyzing drug combination dose-response matrix data. Bioinformatics.

[24] He L, Kulesskiy E, Saarela J, Turunen L, Wennerberg K, Aittokallio T, Tang J (2018). Methods for high-throughput drug combination screening and synergy scoring. Methods Mol Biol.

[25] Yadav B, Wennerberg K, Aittokallio T, Tang J (2015). Searching for drug synergy in complex dose-response landscapes using an interaction potency model. Comput Struct Biotechnol J.

[26] Tang J, Wennerberg K, Aittokallio T (2015). What is synergy? The Saariselkä agreement revisited. Front Pharmacol.

[27] Malyutina A, Majumder MM, Wang W, Pessia A, Heckman CA, Tang J (2019). Drug combination sensitivity scoring facilitates the discovery of synergistic and efficacious drug combinations in cancer. PLoS Comput Biol.

[28] Lee DY, Staddon AP, Shabason JE, Sebro R (2019). Phase I and phase II clinical trials in sarcoma: implications for drug discovery and development. Cancer Med.

[29] Kolb EA, Gorlick R, Reynolds CP, Kang MH, Carol H, Lock R, Keir ST, Maris JM, Billups CA, Desjardins C (2013). Initial testing (stage 1) of Eribulin, a novel tubulin binding agent, by the pediatric preclinical testing program. Pediatr Blood Cancer.

[30] Yahiro K, Matsumoto Y, Fukushi JI, Kawaguchi KI, Endo M, Setsu N, IIda K, Fukushima S, Nakagawa M, Kimura A, et al. Class III β-Tubulin overexpression induces chemoresistance to Eribulin in a leiomyosarcoma cell line. Anal Cell Pathol 2018;2018:1-18. doi:10.1155/2018/8987568. 10.1155/2018/8987568 PMC6033248 30034996

[31] Hayasaka N, Takada K, Nakamura H, Arihara Y, Kawano Y, Osuga T, Murase K, Kikuchi S, Iyama S, Emori M (2019). Combination of eribulin plus AKT inhibitor evokes synergistic cytotoxicity in soft tissue sarcoma cells. Sci Rep.

[32] De Vita A, Miserocchi G, Recine F, Mercatali L, Pieri F, Medri L, Bongiovanni A, Cavaliere D, Liverani C, Spadazzi C (2016). Activity of Eribulin in a primary culture of well-differentiated/dedifferentiated adipocytic sarcoma. Molecules.

[33] Dumontet C, Jordan MA (2010). Microtubule-binding agents: a dynamic field of cancer therapeutics. Nat Rev Drug Discov.

[34] Takezaki Y, Namikawa T, Koyama T, Munekage E, Munekage M, Maeda H, Kitagawa H, Hanazaki K (2016). Antitumor effects of Eribulin Mesylate in gemcitabine-resistant pancreatic cancer cell lines. Anticancer Res.

[35] Kawano S, Asano M, Adachi Y, Matsui J (2016). Antimitotic and non-mitotic effects of Eribulin Mesilate in soft tissue sarcoma. Anticancer Res.

[36] Sampson VB, Vetter NS, Zhang W, Patil PU, Mason RW, George E, Gorlick R, Kolb EA (2016). Integrating mechanisms of response and resistance against the tubulin binding agent Eribulin in preclinical models of osteosarcoma. Oncotarget.

[37] Goto W, Kashiwagi S, Asano Y, Takada K, Takahashi K, Fujita H, Takashima T, Shibutani M, Amano R, Tomita S (2019). The effects of Eribulin on breast cancer microenvironment identified using Eribulin-resistant breast cancer cell lines. Anticancer Res.

[38] Watanabe K, Yui Y, Sasagawa S, Suzuki K, Kanamori M, Yasuda T, Kimura T (2019). Low-dose eribulin reduces lung metastasis of osteosarcoma. Oncotarget.

[39] Edmondson R, Broglie JJ, Adcock AF, Yang L (2014). Three-dimensional cell culture systems and their applications in drug discovery and cell-based biosensors. Assay Drug Dev Technol.

[40] Monderer D, Luseau A, Bellec A, David E, Ponsolle S, Saiagh S, Bercegeay S, Piloquet P, Denis MG, Lodé L (2013). New chondrosarcoma cell lines and mouse models to study the link between chondrogenesis and chemoresistance. Lab Invest.

[41] Gris-Oliver A, Ibrahim YH, Rivas MA, García-García C, Sánchez-Guixé M, Ruiz-Pace F, Viaplana C, Pérez-García JM, Llombart-Cussac A, Grueso J (2021). PI3K activation promotes resistance to eribulin in HER2-negative breast cancer. Br J Cancer.

[42] Zaal EA, Berkers CR (2018). The influence of metabolism on drug response in cancer. Front Oncol.

[43] Perez M, García-Heredia JM, Felipe-Abrio B, Muñoz-Galván S, Martín-Broto J, Carnero A (2020). Sarcoma stratification by combined pH2AX and MAP17 (PDZK1IP1) levels for a better outcome on doxorubicin plus olaparib treatment. Signal Transduct Target Ther.

[44] Zheng S, Aldahdooh J, Shadbahr T, Wang Y, Aldahdooh D, Bao J, Wang W, Tang J (2021). DrugComb update: a more comprehensive drug sensitivity data repository and analysis portal. Nucleic Acids Res.

[45] Bernard M, Cardin GB, Cahuzac M, Ayad T, Bissada E, Guertin L, Bahig H, Nguyen-Tan PF, Filion E, Ballivy O (2020). Dual inhibition of autophagy and PI3K/AKT/MTOR pathway as a therapeutic strategy in head and neck squamous cell carcinoma. Cancers (Basel).

[46] Margue C, Philippidou D, Kozar I, Cesi G, Felten P, Kulms D, Letellier E, Haan C, Kreis S (2019). Kinase inhibitor library screening identifies synergistic drug combinations effective in sensitive and resistant melanoma cells. J Exp Clin Cancer Res.

[47] Miyamoto S, Kakutani S, Sato Y, Hanashi A, Kinoshita Y, Ishikawa A (2018). Drug review: Pazopanib. Jpn J Clin Oncol.

[48] van der Graaf WT, Blay JY, Chawla SP, Kim DW, Bui-Nguyen B, Casali PG, Schöffski P, Aglietta M, Staddon AP, Beppu Y (2012). Pazopanib for metastatic soft-tissue sarcoma (PALETTE): a randomised, double-blind, placebo-controlled phase 3 trial. Lancet.

