# Supplementary material for: Eribulin activity in soft tissue sarcoma monolayer and three-dimensional cell line models: could the combination with other drugs improve its antitumoral effect?
Source: Cancer Cell Int. 2021 Dec 4;21:646. doi: 10.1186/s12935-021-02337-5 (PMC8642967; doi:10.1186/s12935-021-02337-5)
Supplement: Supplementary file 1 — Additional file 1: Table S1. Detailed experimental conditions for employed cell lines, covering seeding densities and drug dose ranges for 2- and 3D conditions. [file 12935_2021_2337_MOESM1_ESM.docx]

**Additional file 1: Table S1.** Detailed experimental conditions for employed cell lines, covering seeding densities and drug dose ranges for 2- and 3D conditions.

| **Cell line** | **Sarcoma subtype** | **Cells per well 2D** | **Cells per well 3D** | **Eribulin dose range 2D (nM)** | **Eribulin dose range 3D (nM)** |
| --- | --- | --- | --- | --- | --- |
| **HT1080** | FS | 4000 | 500 | 10-0.04 | 200-0.78 |
| **SK-UT-1** | LMS | 3000 | 1500 | 10-0.04 | 10-0.04 |
| **SW872** | PLPS | 3000 | 1000 | 10-0.04 | 200-0.78 |
| **LIPODL221** | MLPS | 2000 | 10000 | 100-0.39 | 200-0.78 |
| **93T449** | WDLPS | 3000 | 12000 | 10-0.04 | 200-0.78 |
| **LPS224** | DDLPS | 4000 | 3000 | 100-0.39 | 200-0.78 |
| **LPS246** | DDLPS | 4000 | 4000 | 100-0.39 | 200-0.78 |
|  |  |  |  |  |  |
| **Cell line** | **Doxorubicin dose range 2D (µM)** | **Ifosfamide dose range 2D (mM)** | **Gemcitabine dose range 2D (nM)** | **Trabectedin dose range 2D (nM)** | **Palbociclib dose range 2D (µM)** |
| **HT1080** | 10-0.04 | 10-0.04 | 100-0.39 | 10-0.04 | 100-0.04 |
| **SK-UT-1** | 10-0.04 | 10-0.04 | 100-0.39 | 10-0.04 | 100-0.04 |
| **SW872** | 10-0.04 | 10-0.04 | 100-0.39 | 10-0.04 | 100-0.04 |
| **LIPODL221** | 10-0.04 | 10-0.04 | 100-0.39 | 10-0.04 | 100-0.04 |
| **93T449** | 10-0.04 | 10-0.04 | 100-0.39 | 10-0.04 | 100-0.04 |
| **LPS224** | 10-0.04 | 10-0.04 | 100-0.39 | 10-0.04 | 100-0.04 |
| **LPS246** | 10-0.04 | 10-0.04 | 100-0.39 | 10-0.04 | 100-0.04 |
| FS: Fibrosarcoma, LMS: Leiomyosarcoma, PLPS: Pleomorphic liposarcoma, MLPS: Mixoid liposarcoma, WDLPS: Well-differentiated liposarcoma, DDLPS: Dedifferentiated liposarcoma. | | | | | |
