# Supplementary material for: Eribulin activity in soft tissue sarcoma monolayer and three-dimensional cell line models: could the combination with other drugs improve its antitumoral effect?
Source: Cancer Cell Int. 2021 Dec 4;21:646. doi: 10.1186/s12935-021-02337-5 (PMC8642967; doi:10.1186/s12935-021-02337-5)
Supplement: Supplementary file 2 — Additional file 2: Figure S1. Eribulin dose response curves for cell lines studied. A representative experiment is shown for A) 2D and B) 3D culture conditions. Red line represents the value for GI50 values. C) GI50 Scatter plots. Cells haves been split in two plots according to their values for a better representation. In 3D experiments, only 4 cell lines are presented since the other 3 are over the tested drug range, and considered as resistant.A representative experiment is shown for A) 2D and B) 3D culture conditions. Red line represents the value for GI50 values. C) GI50 Scatter plots. Cells have been split in two plots according to their values for a better representation. In 3D experiments, only 4 cell lines are presented since the other 3 are over the tested drug range, and considered as resistant. [file 12935_2021_2337_MOESM2_ESM.pdf]

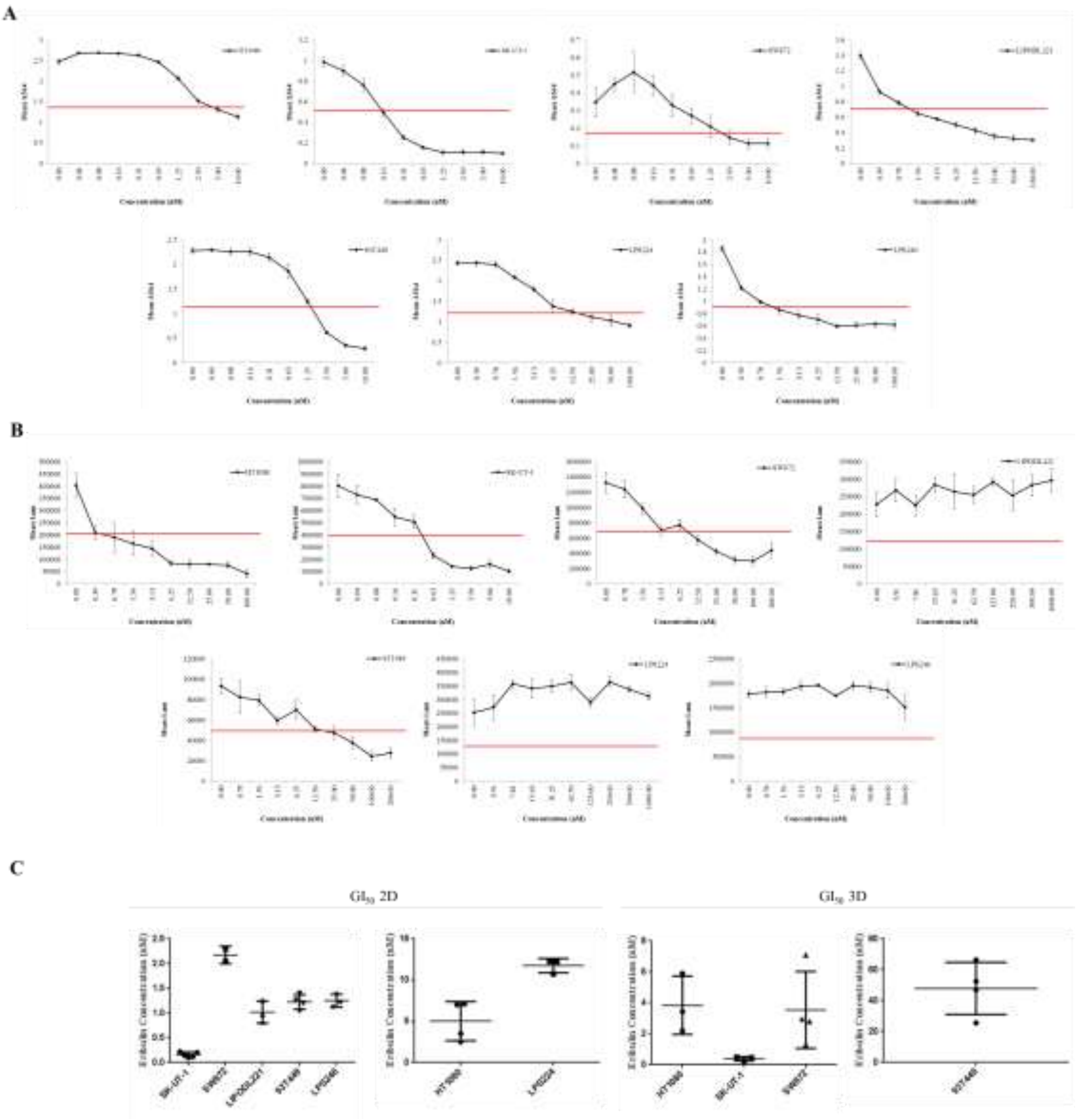

Supplementary Figure 1. Eribulin dose response curves for cell lines studied. A representative experiment is shown for A) 2D and B) 3D culture conditions. Red line represents the value for GI_{50} values. C) GI_{50} Scatter plots. Cells have been split in two plots according to their values for a better representation. In 3D experiments, only 4 cell lines are presented since the other 3 are over the tested drug range, and considered as resistant.
