# Supplementary material for: Eribulin activity in soft tissue sarcoma monolayer and three-dimensional cell line models: could the combination with other drugs improve its antitumoral effect?
Source: Cancer Cell Int. 2021 Dec 4;21:646. doi: 10.1186/s12935-021-02337-5 (PMC8642967; doi:10.1186/s12935-021-02337-5)
Supplement: Supplementary file 3 — Additional file 3: Figure S2. Eribulin effect on morphology. Phenotypical changes of cell lines exposed to eribulin for 72h. [file 12935_2021_2337_MOESM3_ESM.pdf]

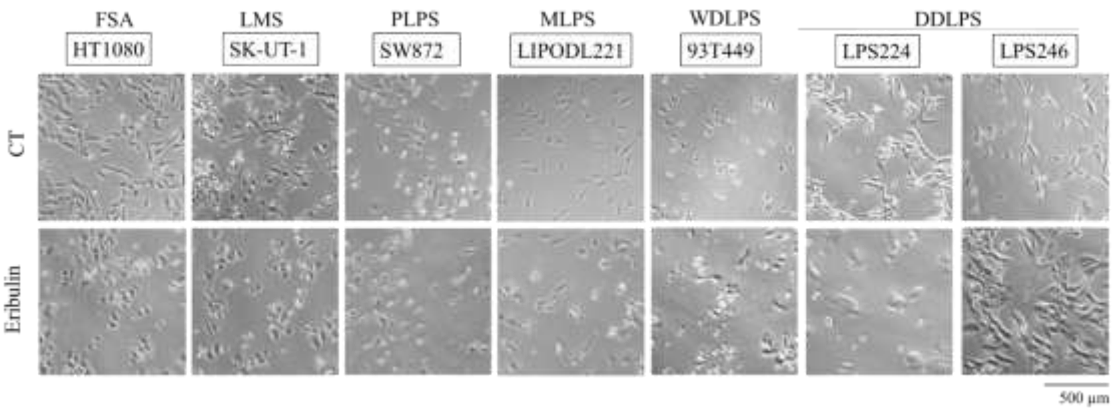

Supplementary Figure 2. Eribulin effect on morphology. Phenotypical changes of cell lines exposed to eribulin for 72h.
