# Supplementary material for: Eribulin activity in soft tissue sarcoma monolayer and three-dimensional cell line models: could the combination with other drugs improve its antitumoral effect?
Source: Cancer Cell Int. 2021 Dec 4;21:646. doi: 10.1186/s12935-021-02337-5 (PMC8642967; doi:10.1186/s12935-021-02337-5)
Supplement: Supplementary file 4 — Additional file 4: Table S2. 2D GI50 values for studied drugs. The concentration value is displayed as mean ± standard deviation. [file 12935_2021_2337_MOESM4_ESM.docx]

**Additional file 4: Table S2**. 2D GI_50_ values for studied drugs. The concentration value is displayed as mean ± standard deviation.

| **Cell line** | **Sarcoma subtype** | **Doxorubicin (nM)** | **Ifosfamide (mM)** | **Gemcitabine (nM)** |
| --- | --- | --- | --- | --- |
| **HT1080** | FS | 306.25 ± 2.48 | 3.99 ± 1.80 | 5.73 ± 2.62 |
| **SK-UT-1** | LMS | 29.00 ± 3.00 | 7.53 ± 2.43 | 4.25 ± 0.79 |
| **SW872** | PLPS | 32.71 ± 7.35 | 4.89 ± 0.80 | 4.66 ± 0.43 |
| **DL221** | MLPS | 100.41 ± 37.94 | 4.05 ± 0.98 | 14.09 ± 3.84 |
| **93T449** | WDLPS | 168.13 ± 69.63 | 5.91 ± 1.45 | 20.24 ± 1.28 |
| **LPS224** | DDLPS | 442.03 ± 36.65 | 5.15 ± 0.72 | 24.32 ± 14.02 |
| **LPS246** | DDLPS | 189.30 ± 72.32 | 6.50 ± 0.34 | 23.48 ± 6.72 |

| **Cell line** | **Sarcoma subtype** | **Trabectedin (nM)** | **Pazopanib (μM)** | **Palbociclib (μM)** |
| --- | --- | --- | --- | --- |
| **HT1080** | FS | 0.62 ± 0.15 | 1.62 ± 0.36 | 20.84 ± 6.41 |
| **SK-UT-1** | LMS | 0.19 ± 0.08 | 9.88 ± 0.96 | 8.61 ± 1.72 |
| **SW872** | PLPS | 0.24 ± 0.06 | 7.78 ± 2.02 | 4.67 ± 1.17 |
| **DL221** | MLPS | 0.26 ± 0.02 | 1.95 ± 0.64 | 2.19 ± 0.21 |
| **93T449** | WDLPS | 0.82 ± 0.34 | 4.43 ± 0.84 | 10.19 ± 0.86 |
| **LPS224** | DDLPS | 0.57 ± 0.15 | 5.32 ± 0.74 | 16.28 ± 2.31 |
| **LPS246** | DDLPS | 0.40 ± 0.06 | 9.80 ± 3.59 | 14.00 ± 1.58 |
| FS: Fibrosarcoma, LMS: Leiomyosarcoma, PLPS: Pleomorphic liposarcoma, MLPS: Mixoid liposarcoma, WDLPS: Well-differentiated liposarcoma, DDLPS: Dedifferentiated liposarcoma. | | | | |
