# Supplementary material for: Eribulin activity in soft tissue sarcoma monolayer and three-dimensional cell line models: could the combination with other drugs improve its antitumoral effect?
Source: Cancer Cell Int. 2021 Dec 4;21:646. doi: 10.1186/s12935-021-02337-5 (PMC8642967; doi:10.1186/s12935-021-02337-5)
Supplement: Supplementary file 5 — Additional file 5: Figure S3. Effect of eribulin in Cell cycle. Bar plots show Cell cycle distribution for subG0, G0/G1, S and G2/M phases comparison in untreated and eribulin exposed cells. *p<0.05, **p<0.005. [file 12935_2021_2337_MOESM5_ESM.pdf]

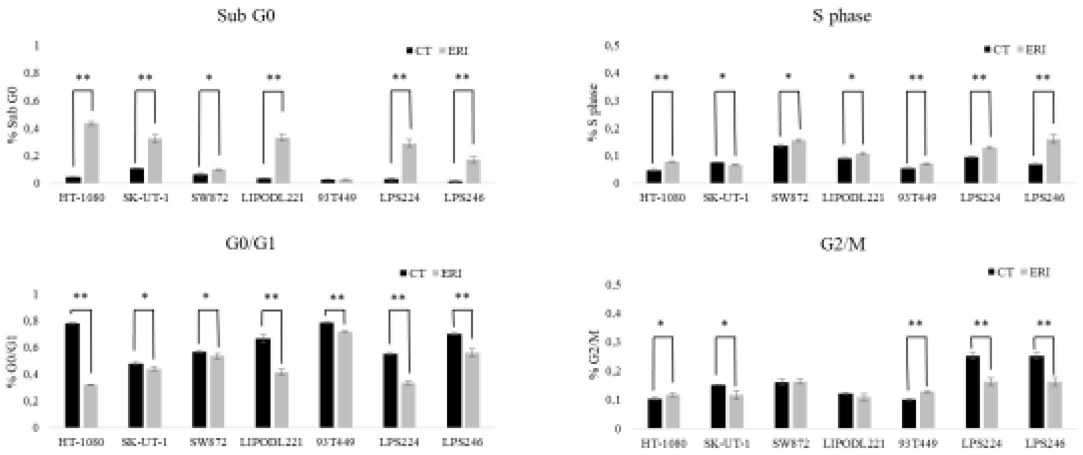

Supplementary Figure 3. Effect of eribulin in Cell cycle. Bar plots show Cell cycle distribution for subG0, G0/G1, S and G2/M phases comparison in untreated and eribulin exposed cells. *p<0.05, **p<0.005.
