# Supplementary material for: Eribulin activity in soft tissue sarcoma monolayer and three-dimensional cell line models: could the combination with other drugs improve its antitumoral effect?
Source: Cancer Cell Int. 2021 Dec 4;21:646. doi: 10.1186/s12935-021-02337-5 (PMC8642967; doi:10.1186/s12935-021-02337-5)
Supplement: Supplementary file 6 — Additional file 6: Figure S4. Pathway enrichment analysis for disregulated processes between parental and resistant 93T449. NES: Normalized enrichment score; pval: p value. [file 12935_2021_2337_MOESM6_ESM.pdf]

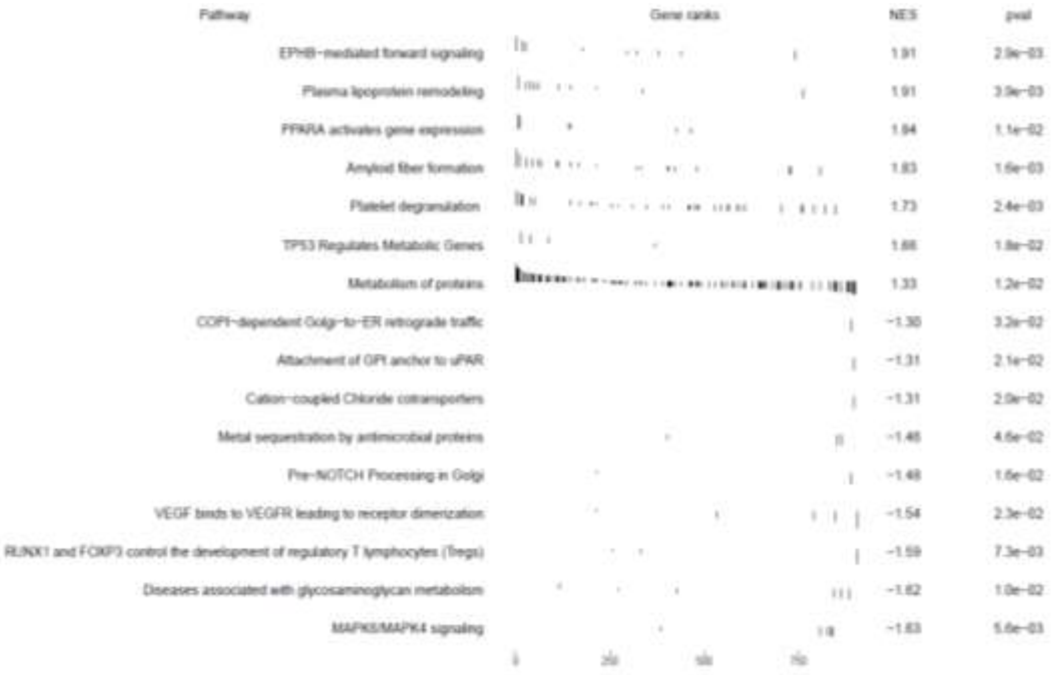

Supplementary Figure 4. Pathway enrichment analysis for disregulated processes between parental and resistant 93T449. NES: Normalized enrichment score; pval: p value.
