# Supplementary material for: Eribulin activity in soft tissue sarcoma monolayer and three-dimensional cell line models: could the combination with other drugs improve its antitumoral effect?
Source: Cancer Cell Int. 2021 Dec 4;21:646. doi: 10.1186/s12935-021-02337-5 (PMC8642967; doi:10.1186/s12935-021-02337-5)
Supplement: Supplementary file 7 — Additional file 7: Figure S5. Tidycomb Analysis. Eribulin combination with A. Ifosfamide, B. Pazopanib and C. Doxorubicin, on LMS and LPS cell lines. The area highlighted in red corresponds to the score values considered as indicators of synergy. [file 12935_2021_2337_MOESM7_ESM.pdf]

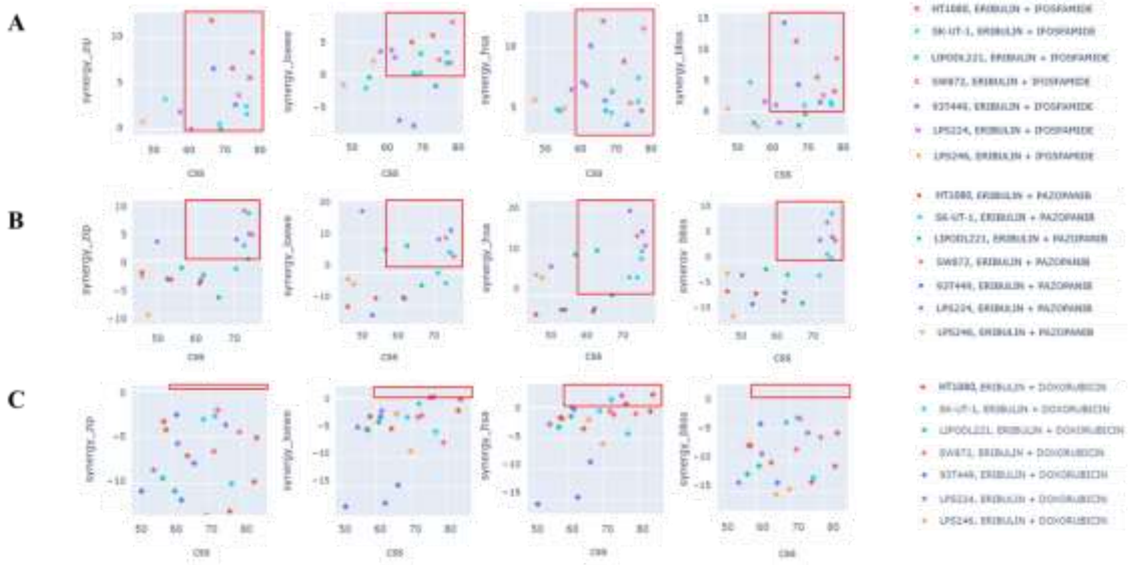

Supplementary Figure 5. Tidycomb Analysis. Eribulin combination with A. Ifosfamide, B. Pazopanib and C. Doxorubicin, on LMS and LPS cell lines. The area highlighted in red corresponds to the score values considered as indicators of synergy.
